# Self-Efficacy Between Previous and Current Mathematics Performance of Undergraduate Students: An Instrumental Variable Approach to Exposing a Causal Relationship

**DOI:** 10.3389/fpsyg.2020.556607

**Published:** 2021-01-18

**Authors:** Yusuf F. Zakariya

**Affiliations:** Department of Mathematical Sciences, University of Agder, Kristiansand, Norway

**Keywords:** self-efficacy, prior mathematics knowledge, undergraduate learning, causal model analysis, instrumental variable approach

## Abstract

**Purpose:**

Self-efficacy has been argued theoretically and shown empirically to be an essential construct for students’ improved learning outcomes. However, there is a dearth of studies on its causal effects on performance in mathematics among university students. Meanwhile, it will be erroneous to assume that results from other fields of studies generalize to mathematics learning due to the task-specificity of the construct. As such, attempts are made in the present study to provide evidence for a causal relationship between self-efficacy and performance with a focus on engineering students following a mathematics course at a Norwegian university.

**Method:**

The adopted research design in the present study is a survey type in which collected data from first-year university students are analyzed using structural equation modeling with weighted least square mean and variance adjusted (WLSMV) estimator. Data were generated using mainly questionnaires, a test of prior mathematics knowledge, and the students’ final examination scores in the course. The causal effect of self-efficacy was discerned from disturbance effects on performance by using an innovative instrumental variable approach to structural equation modeling.

**Results:**

The findings confirmed a significant direct effect of the prior mathematics knowledge test (β = 0.52, SE = 0.01, *p* < 0.001) on self-efficacy, a significant direct effect (β = 0.43, SE = 0.19, *p* = 0.02) of self-efficacy on performance, and a substantial mediating effect (β = 0.22, SE = 0.10, *p* = 0.03) of self-efficacy between a prior mathematics knowledge test and performance. Prior mathematics knowledge and self-efficacy explained 30% variance of the performance. These findings are interpreted to be substantial evidence for the causal effect of self-efficacy on students’ performance in an introductory mathematics course.

**Conclusion:**

The findings of the present study provide empirically supports for designing self-efficacy interventions as proxies to improve students’ performance in university mathematics. Further, the findings of the present study confirm some postulates of Bandura’s agentic social cognitive theory.

## Introduction

There has been a growing interest in research on students’ affective factors and their contributions to learning outcomes at all levels of education. Apart from the fact that some of these affective factors, e.g., self-efficacy, satisfactorily predict students’ performance, an explanation for the growing interest may be ascribed to the ease of developing interventions that influence such factors (Czocher et al., 2019). For instance, perceived self-efficacy, which has been conceptualized as “beliefs in one’s capabilities to organize and execute the courses of action required to produce given attainments” (Bandura, 1997, p. 3), was shown to predict academic achievement better than intelligence test scores, measures of self-esteem, and personal traits among school children (Zuffianò et al., 2013; Özcan and Eren Gümüş, 2019). With regards to the learning outcomes in undergraduate mathematics, perceived self-efficacy was found to be a better predictor of performance than the usefulness of mathematics, prior mathematics knowledge, self-concept (Pajares and Miller, 1994), mathematics anxiety, and mental ability (Pajares and Kranzler, 1995). A high sense of self-efficacy has also been linked with the adoption of deep approaches to learning, high learning motivation, positive attitude toward mathematics. In contrast, a low sense of self-efficacy has been linked with the adoption of surface approaches to learning, high mathematics anxiety, and low interest in mathematics (Bandura, 1997; Rozgonjuk et al., 2020; Zakariya et al., 2020b). More recently, Schukajlow et al. (2019) demonstrate an approach through which constructing multiple solutions to real-life problems can be used as an intervention to influence students’ self-efficacy in mathematics. Student-centered instructional methods have also been linked with high self-efficacy (Lahdenperä et al., 2019).

Even though the relationship between self-efficacy and students’ performance has been widely studied, little is known about the causal effect of the former on the latter as it concerns the learning of university mathematics. The available studies on self-efficacy with a focus on university mathematics are either relatively old (e.g., Hackett and Betz, 1989; Pajares and Miller, 1994), utilized regression models which make it difficult to evaluate causal hypotheses between self-efficacy and students’ performance in mathematics (e.g., Peters, 2013), or do not account for confounding factors in their structural models (e.g., Roick and Ringeisen, 2018). By a causal effect, the author means, if A is a cause of B then at least all the following conditions are satisfied: (1) A temporarily precedes B, i.e., data on A are collected before data on B or A is theorized to happen before B; (2) There is a substantial correlation between A and B; (3) There should not be a third variable C that explains the relationship between A and B (Antonakis et al., 2010). The third condition is the most difficult to meet, especially in non-experimental research. Such variable C will always exist. The most important question is how well a researcher can control it? Among the several attempts that have been shown empirically to yield satisfactory performance in controlling for an extraneous variable, such as C in non-experimental research, is the use of instrumental variable approach (Antonakis et al., 2010; Bollen, 2019). The basic idea of the instrumental variable approach is to find a fourth variable called an *instrument* that satisfies some properties (which will be explained in the “Materials and Methods” section) and use it to discern the actual effect of A on B from any confounding effects of C (Greenland, 2000; Bollen, 2019).

As such, the primary purpose of the present study is to investigate the causal effects of perceived self-efficacy on the current students’ performance in mathematics among engineering students with an application of the innovative instrumental variable approach to modeling. Further, the effects of prior mathematics knowledge on the perceived self-efficacy and the current students’ performance are also investigated. An advantage of using the innovative instrumental variable approach in exposing these causal effects lies in a fact that reliable estimates of effects can be justified. Despite the wide application of the instrumental variable approach among epidemiologists and econometricians (Antonakis et al., 2010), it is innovative in the present study because the author is not aware of its previous use in mathematics education research. It is the opinion of the author that policymakers, researchers, and education stakeholders are more interested in studies that explore answers to questions on what brings about improved students’ performance and to what extent? Rather than, in studies that focus on correlations between variables whose findings are either complicated to interpret or beset by unclear conclusions (Pajares and Miller, 1994). The present study, therefore, attempts to address the following research question: What are the direct and indirect causal effects of prior mathematics knowledge and perceived self-efficacy on performance in mathematics among engineering students? The author draws on both theoretical and analytical perspectives to address this question. The statistical analyses in the present article are moderately advanced and up to date. However, the author has deliberately chosen a simple language of presentation with less mathematical abstractions to make the findings more accessible.

The remaining part of the present article is organized as follows: An overview of a theoretical perspective which leads to the formulation of research hypotheses is presented in the next section. Next is the “Materials and Methods” section where research methodological related issues are presented. The fourth section presents analyses and results. The major findings are discussed in the fifth section, including potential limitations and recommendations for further studies. Finally, the article closes with some remarks.

## Conceptual Framework

Perceived self-efficacy is firmly rooted in the agentic social cognitive theory (henceforth, social cognitive theory) as propagated by Albert Bandura in his decades of work on the theory (Bandura, 2001, 2012). Bandura, dissatisfied with some ontological and epistemological claims of traditional cognitive theory (cognitive theory), developed the social cognitive theory. The ontological paradigm shift from the cognitive theory lies in a rejection of dualism between personal agent and object of actions. Reciprocal determinism is an epistemological position that differentiates the social cognitive theory from the cognitive theory. Reciprocal determinism is a feedback causal model of the relationship between behavioral factors, personal factors, and environmental factors (Bandura, 2012). That is, an individual’s behavioral changes are consistently being regulated and modified by interacting with social factors in the environment whose feedback influences the next actions and outcomes.

Therefore, it is argued that perceived self-efficacy being an integral part of the personal factors cannot be a fixed trait. It changes in response to changes that occur to the rest of the factors in the reciprocal deterministic system (Bandura, 2012). As it concerns mathematics learning, Borgonovi and Pokropek (2019) conceptualized and described reciprocal determinism as “the sets of relationships underlying the interactions between (a) individuals’ exposure to mathematics tasks, (b) mathematics self-efficacy beliefs, and (c) mathematics ability” (p. 269). Therefore, it follows logically to argue that mathematics perceived self-efficacy (henceforth, self-efficacy) is a task-specific construct and affects the performance of engineering students in calculus tasks. Earlier studies have investigated the task-specificity of self-efficacy and confirm that proper attention to task-specificity is a satisfactory way to improve the predictive power of self-efficacy on students’ performance in mathematics (Pajares and Miller, 1995). In the present study, the implications of the task-specificity of self-efficacy go beyond the prediction of performance but extend to the research focus and adoption of a self-efficacy measure whose detail is presented in the “Materials and Methods” section.

The concept of self-efficacy has emerged from the social cognitive theory to become a theory on its own. According to the self-efficacy theory, there are four primary sources of self-efficacy beliefs: *enactive mastery experience*, i.e., personal previous task-based achievement, *vicarious experience*, i.e., experience gained by monitoring peers or people around, *verbal/social persuasions*, i.e., complementary or contradictory feedback received from others, and *physiological or affective states*, i.e., physical or emotional situations during the behavioral changes (Bandura, 2008). Among the sources of influence of self-efficacy, previous task-based achievement has been shown empirically to have the most significant impact on students’ self-efficacy on mathematics tasks (e.g., Joët et al., 2011; Zientek et al., 2019). Further, Yurt (2014) showed that, apart from predicting self-efficacy, mastery experience has a highly significant correlation with students’ mathematics achievement as measured by the end of the semester course grades. As such, if pre-university mathematics content knowledge is considered to be part of the personal previous task-based achievement, then a causal effect is expected between prior mathematics knowledge and the self-efficacy of engineering students. Therefore, the following hypothesis is formulated:

*Hypothesis one:* There is a direct effect of prior mathematics knowledge on self-efficacy among first-year engineering students.

Fundamental goals of self-efficacy theory within the teaching and learning context are to explain, predict and evaluate differences in students’ performance that are brought about by their self-efficacy (Bandura, 2012). A high sense of self-efficacy instills confidence on students’ minds when confronted with difficult and challenging mathematical tasks and as such, enables the students to persevere, so that desired outcomes are achieved. In contrast, students with a low sense of self-efficacy cannot forebear difficult situations, doubt their ability, and as such, perform poorly on the learning material. Roick and Ringeisen (2018) reported a longitudinal study in which the contribution of self-efficacy to students’ performance in mathematics was investigated. They used a structural equation modelling (SEM) approach with a sample of 206 university students and found that self-efficacy predicts students’ performance. Similar corroborative findings on the predictive power of self-efficacy as it concerns university mathematics can be found, elsewhere (e.g., Pajares and Miller, 1994; Pajares and Kranzler, 1995). However, as it is highlighted in the introduction section of the present article, some of these studies have one limitation or the other that makes it difficult to deduce substantial causal claims between self-efficacy and students’ performance in mathematics. More so, it could be erroneous to assume that findings from other fields generalize to the university mathematics context considering the task-specificity of self-efficacy. Instead, the author draws on these studies and some postulates of self-efficacy theory to formulate the following hypotheses:

*Hypothesis two*: There is a direct effect of self-efficacy on engineering students’ performance in a first-year calculus course.
*Hypothesis three*: Self-efficacy mediates the effect of engineering students’ prior mathematics knowledge on their performance in a first-year calculus course.

## Materials and Methods

### Research Focus

The present study focuses on the engineering students following a first-year mathematics course at a Norwegian university. Students enrolled in a first-year mathematics course are chosen as participants in the present study for several reasons. First, the author can assess their pre-university mathematics knowledge effectively better than that of students in year two, year three and year four. Second, they are more susceptible to poor performance, high anxiety, and lack of confidence due to their transition from secondary school to university and newness to the university culture. In line with the task-specificity of self-efficacy, data collected from students enrolled on a common mathematics course are more likely to be objective and when analyzed could give a close estimation of the causal relationship between the research constructs. Further, engineering students are the target group in the present study because they form the largest student population following a common mathematics course in the university.

### Sample of the Study

An effective sample of 189 engineering students voluntarily participated in the study, most of whom are men (75%). Their age distributions are as follows: 17–20 years (31%), 21–25 years (49%), 26–35 years (15%), and over 36 years (5%). The inclusion and exclusion criteria are based on voluntary consent. As such, the sample can be characterized as a convenient sample. The language of instruction in the course is Norwegian as well as the language used for the mandatory exercises and examinations.

### Measures

#### Prior Mathematics Knowledge

The author adopted a Norwegian mathematics test as a proxy to expose the prior mathematics content knowledge of the participating students in the present study. The test was designed by the Norwegian Mathematical Council to assess pre-university mathematics content knowledge, and it is administered every two years, independent of the present study, to first-year students across several universities and colleges in Norway. It is a 22-item test in which questions are formulated based on the secondary school curriculum. It is assumed that the test is most appropriate in the present study because it has been developed within the Norwegian context and consistently been applied to serve a similar purpose as that of the present study, for the past three decades. Further, the construct validity and the reliability index (using Omega coefficient) of the test have been investigated using a latent variable approach in Mplus 8.3 program, and the latter was found to be 0.92 which together with the unidimensionality of the test show high internal consistency of its items (Zakariya et al., 2020a). However, only a portion of the test (17 items, henceforth, PKMT – prior knowledge of mathematics test) that is of high psychometric properties such as appropriate item difficulty indices (−2.795 to 0.923), item discrimination indices (0.421–1.354), item reliability (0.151–0.646), and unidimensionality, i.e., all the 17 items expose a common latent construct (Zakariya et al., 2020a), is used in the present study. The 17-item PKMT has only two standard multiple-choice questions, and the remaining 15 questions require short answers. All the questions examine the basic knowledge of operations with fractions, decimals, percentages, ratios, similar triangles, speed and distance, and some word problems. A score of 1 point was assigned to a correct answer and a 0 point, otherwise.

#### Calculus Self-Efficacy

Following the task-specificity of the self-efficacy, the calculus self-efficacy inventory (CSEI) was adopted in the present study. The CSEI was developed with a specific purpose of exposing students’ self-efficacy in solving some mathematical tasks drawn from the first-year introductory calculus course (Zakariya et al., 2019). According to the self-efficacy theory, such an inventory offers the best precision in exposing the construct (Bandura, 2006). The CSEI has two parts: preliminary and main parts. The preliminary part of the CSEI contains questions on gender, age, and grade points of students in the highest upper secondary school mathematics course (HGP) they followed before their enrollment into the university. Responses of students to the question on HGP, in addition to the PMKT, are used as proxies to measure their prior mathematics content knowledge. The response values on this item ranging from 1 to 6 points depending on the grades. Further, the main part of the CSEI contains 13 items on exam-type mathematics tasks in which the contents are drawn from the current course curriculum followed by the students. The responses of students on this part of CSEI are used as proxies to expose the latent construct of self-efficacy. The students rate their confidence, on a scale of 0–100, in their belief that they can successfully solve the mathematics tasks. The conceptualization, operationalization, and psychometric properties of the CSEI have been previously studied using factor analysis in FACTOR program coupled with Spearman’s rank correlation and well documented (Zakariya et al., 2019). The CSEI was found to possess construct and discriminant validity, unidimensionality, and with a reliability index of 0.90 using ordinal coefficient alpha (Zakariya et al., 2019).

#### Performance

Finally, the current performance of students in the present study is operationalized and measured by their final scores achieved in the first-year introductory calculus course they followed. It is presumed in the present study, and consistent with the literature (e.g., Cano et al., 2018), that such scores offer the best opportunity to compare individual performance in the course.

### Data Collection and Ethical Considerations

The data used in the present study are collected mainly through an online platform, SurveyXact. The author together with his research team independently converted the PKMT to an online test after being granted permission to access the test by the Norwegian Mathematical Council. Similarly, an online version of the CSEI was also prepared. The students were informed of the purpose of the study at a class visit before data collection. Their voluntary consent to take part in the study was sought. As such, they were promised of no consequence, whatsoever, for anyone who decides not to participate in the study. The students were informed that their data will be treated with a high level of security and confidentiality in line with the regulations of the Norwegian Centre for Research Data.

The data were collected on three occasions. At the first occasion, the PKMT was administered in which 40 min of class time was used on the test. This test administration took place in the early weeks of the Autumn semester 2019 because the beginning of the semester is the best time to assess pre-university mathematics content knowledge. On the second occasion, toward the last week of lectures in the Autumn semester 2019, the researchers administered the CSEI through students’ registered emails with the university. Because items of the CSEI are drawn from the ongoing mathematics course curriculum, the administration of CSEI was deliberately delayed until the end of the semester. This delay was aimed at ensuring a substantial part of the course curriculum had been covered. The collected data from the two occasions were merged to form an effective sample for the study. In order to ensure the personal data protection regulations are met, the students’ administrative affairs office was involved in the process when it came to collating identifiable data. The researcher simply sent the generated survey data to the examination office where the individual final examination scores in the course were added. Afterward, the examination office removed any identifiable information from the data set, and the researcher was provided with a completely anonymized data set. This procedure constitutes the third occasion of the data collection. The data were screened for out of range values, missing values, and normal distribution, all of which pose no challenge to the analyses.

### Data Analysis

#### The Hypothesized Model and Choice of an Instrument

**FIGURE 1:**
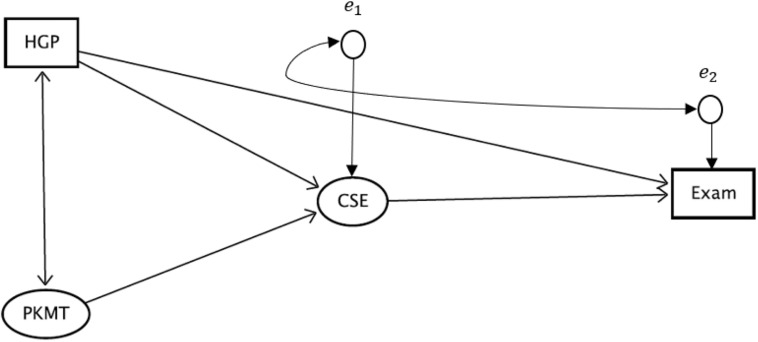
The hypothesized model of the relationship between prior mathematics knowledge, self-efficacy, and students’ performance in an introductory calculus course. Both HGP and PKMT are measures of the prior mathematics knowledge of the students, CSE is a measure of the self-efficacy, and Exam represents a measure of performance. The items of both PKMT and CSE are not included in Figure 1 to enhance the readability of the figure.

The hypothesized model of the relationship between the calculus self-efficacy (CSE), prior mathematics knowledge (HGP and PKMT), and students’ performance in the course (Exam) is presented in Figure 1. The main aim of evaluating this model is to estimate the effects of CSE and HGP on Exam. However, there is a challenge with the model. This is because there are some omitted variables, such as the similarity between items on the CSEI and the final examination. The omitted variables act as common causes of both the CSE and the Exam, thereby causing the errors *e*_*1*_ and *e*_*2*_ to correlate. This correlation may bias the estimate of the effect of self-efficacy on performance, and thereby constitutes an endogeneity problem in the model (Antonakis et al., 2010). CSE is an endogenous variable in the model because both HGP and PKMT predict it, and it predicts Exam. A way to circumvent this problem, so that a reliable estimate of the effect of self-efficacy on performance can be found is to introduce an instrumental variable, simply called an *instrument*, in the model (Greenland, 2000). It is assumed that the omitted variables do not affect both HGP and PKMT because they are exogenous variables, i.e., they are not predicted by any variable in the model, and as such do not need an instrument. The double-headed arrow between HGP and PKMT in Figure 1 is a standard notation for correlation between the variables in the SEM literature. It should not be confused with a feedback effect.

An *instrument* “I” is an exogenous variable that satisfies the following properties: (a) “I” has a direct effect on the endogenous variable (CSE) that needs an instrument; (b) The direct effect of “I” on the outcome variable (Exam) is close to zero or completely negligible in the presence of the endogenous variable; (c) “I” should not correlate with the errors associated with the outcome variable (Greenland, 2000; Antonakis et al., 2010). The preliminary analysis in the present study shows that PKMT is the only variable that satisfies the properties (a)–(c), and thus, it was selected as an instrument to discern the true effect of self-efficacy on the performance from the omitted causes in the model.

#### The Procedure of Data Analysis

The collected data are analyzed using the SEM approach to evaluate the model presented in Figure 1 and as such, to confirm the plausibility of the research hypotheses. The SEM approach was adopted in the present study because it offers the best and most robust modeling capacity to evaluate causal hypotheses (Bollen and Pearl, 2013). SEM does it better than the path analysis, multiple linear regression, and the partial-least square techniques (Antonakis et al., 2010). Because PKMT was dichotomously scored, the weighted least square mean and variance adjusted (WLSMV) estimator was used which has been shown to provide satisfactory parameter estimates in the analysis of categorical data (Suh, 2015). The author ascertains the “data fitness” of the hypothesized model by looking at both global and local fit indices and parameters. The global fit criteria used are chi-square ratio to the degree of freedom of less than 3, comparative fit (CFI) and Tucker-Lewis indices of greater than or close to 0.90 (Bentler, 1990), and a root mean square error of approximation (RMSEA) value of less than 0.08 (Brown, 2015). The local fits of the model parameters are ascertained by looking at the magnitude and the significant levels of factor loadings, standard errors, and the residual variance, in line with the best practice in SEM literature (Marsh et al., 2004). All the analyses were performed in Mplus 8.3 program.

## Results

The first set of results are from the evaluations of one-factor models for each of the prior mathematics knowledge test and the calculus self-efficacy measurement models. These measurement models are evaluated separately before an evaluation of the hypothesized structural model. In this way, the author could detect and correct any local misspecification in each of the measurement models. This two-step of measurement-before-structural model evaluation has been proven efficient and highly recommended in SEM literature (Byrne, 2012). The dichotomously scored 17 items of the PKMT are hypothesized to expose a common latent factor (prior mathematics knowledge) and tested. All the factor loadings are freely estimated, and the factor variance is fixed to 1 so that the model is identified (Zakariya et al., 2020a). Similarly, the 13 items of the CSEI are hypothesized to expose a common latent factor (self-efficacy) and tested. The factor loadings are freely estimated, the factor variance is fixed to 1, two error covariances between item 09 and item 11 as well as between item 12 and item 13 are allowed in the model as recommended by Zakariya et al. (2019). Further, a maximum likelihood with robust standard errors (MLM) estimator was used instead of the WLSMV because the students’ responses on the CSEI are continuous and not categorical. The results from these analyses with regards to the selected global fit indices are presented in Table 1.

**TABLE 1:** The selected global fit indices for evaluated PKMT and CSEI measurement models.

Global fit indices	PKMT model	CSEI model
**Chi-square**		
Estimate (χ^2^)	143.793	132.162
Degrees of freedom (*df*)	119	64
χ^2^/*df*	1.21	2.065
**CFI/TLI**		
CFI	0.944	0.911
TLI	0.936	0.892
**RMSEA**		
Estimate	0.043	0.076
90 percent confidence interval	[<0.001, 0.066]	[0.057, 0.094]
Probability RMSEA ≤ 0.05	0.676	0.013

**FIGURE 2:**
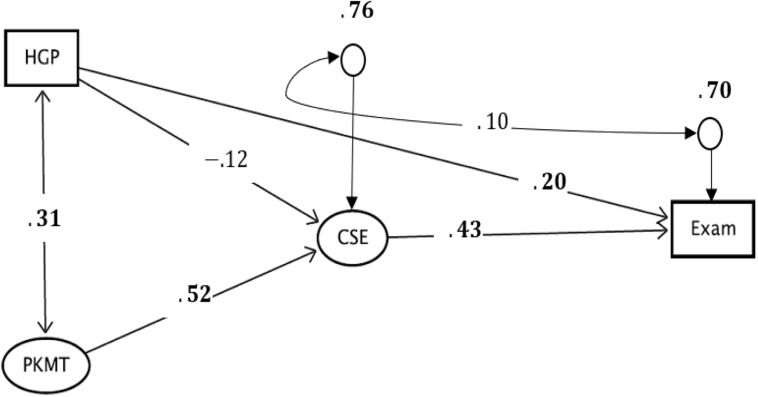
The evaluated model of the relationship between prior mathematics knowledge, self-efficacy, and students’ performance in an introductory calculus course. Both HGP and PKMT are measures of the prior mathematics knowledge of the students, CSE is a measure of the self-efficacy, and Exam represents a measure of performance. The significant estimates are in bold faces, and the items of both PKMT and CSE are not included in Figure 1 to enhance the readability of the figure. The full figure that contains all the items and the associated model parameters is available in Appendix Figure A1.

The results presented in Table 1 show that the global fit indices are within the recommended ranges for acceptable model fits of the analyzed data. In particular, the ratios of chi-square values to the degrees of freedom, the CFI and the TLI values suggest an acceptable fit for both the PKMT and CSEI models. The RMSEA value and its associated 90% confidence interval with a non-significant *p*-value of the PKMT model show that there is an excellent agreement between the model and the data (Bentler, 1990). Even though the *p*-value of the 90% confidence interval for the RMSEA value in CSEI model is significant, the estimate is lower than 0.08, which suggests a good fit (Brown, 2015). The factor loadings are significant and moderately high, the standard and residual errors are low which are suggestive of acceptable local fit statistics for both the PKMT and CSEI models (Marsh et al., 2004). As such, the author proceeds to the evaluation of the hypothesized structural model, as presented in Figure 1, and the resulting global fit indices are presented in Table 2. Further, Figure 2 presents the standardized estimates of the causal effects between the research variables.

**TABLE 2:** The selected global fit indices of the evaluated hypothesized structural model of the relationship between the research variables.

Global fit indices	Hypothesized model (Figure 1)
**Chi-square**	
Estimate (χ^2^)	492.432
Degrees of freedom (*df*)	458
χ^2^/*df*	1.075
**CFI/TLI**	
CFI	0.958
TLI	0.954
**RMSEA**	
Estimate	0.020
90 per cent confidence interval	[<0.001, 0.033]
Probability RMSEA ≤ 0.05	1.000

The results presented in Table 2 show an excellent model fit of the evaluated hypothesized structural relationship between the research variables. An excellent model fit in the sense that there is a substantial agreement between the hypothesized model and the analyzed data. This model fit can be deduced from the selected global fit indices that are within the recommended ranges. The ratio of chi-square estimate to the degree of freedom is far less than 3. The CFI and TLI indices are greater 0.95, which indicate an excellent model fit according to the cutoff criteria by Hu and Bentler (1999). The RMSEA estimate together with its perfect (*p*-value = 1.000) 90% confidence interval, suggested that there is a substantial-close fit between the model and analyzed data (Brown, 2015). The global fit indices presented in Table 2 strengthen the plausibility of the standardized estimates of the causal effects presented in Figure 2.

The results presented in Figure 2 show reliable estimates of the standardized causal effects between the research variables. The reliability of these estimates has been strengthened by the excellent global fit indices reported in Table 2. Figure 2 shows a significant direct effect of PKMT (β = 0.52, standard error – SE = 0.01, *p* < 0.001) on self-efficacy. The direct effect of HGP on self-efficacy is negative and not significant (β = −0.12, SE = 0.09, *p* > 0.05). Even though, one would have expected a positive effect of HGP on self-efficacy given that students with high grade points in upper secondary school mathematics are expected to have high self-efficacy. The result of the present study does not conform to this expectation. These results show that among the two measures of prior mathematics knowledge, it is only the scores of students on the pre-university mathematics test that have a substantial effect on students’ self-efficacy. As such, Hypothesis one is confirmed. The correlation between PKMT and HGP is significant (*r* = 0.31, SE = 0.08, *p* < 0.001), and it is expected. This is because both PKMT and the HGP are hypothesized to expose different facets of a construct. The correlation between these variables was evaluated instead of a causal relationship for two reasons. The first reason is that they expose different facets of a construct while the second reason is to comply with the recommendations of instrumental variable approach for handling endogeneity problem due to omitted variables in the model (e.g., Kenny, 2012).

It is also revealed in Figure 2 that the direct effect of self-efficacy on students’ performance is significant (β = 0.43, SE = 0.19, *p* = 0.02) and a significant standardized residual estimate of 0.76. These results confirm the plausibility of Hypothesis two. The residual error shows that the prior mathematics knowledge of students explains 24% of the factor variance in self-efficacy. The percentage of the explained factor variance is moderate, considering the limited number of variables that predict self-efficacy in the model. The error covariance between the self-efficacy and students’ performance is not significant (*r* = 0.10, SE = 0.25, *p* > 0.05) which is a good result as it confirms the reliability of the estimated effect of self-efficacy on performance after introducing the instrument in the model. Figure 2 also shows that the direct effect of HGP on the students’ performance is significant (β = 0.20, SE = 0.07, *p* = 0.005).

More so, the results of the mediation analysis show the standardized total effect of prior mathematics knowledge (PKMT and HGP) on performance to be 0.37. A significant indirect effect of PKMT through self-efficacy was found (β = 0.22, SE = 0.10, *p* = 0.03), and a non-significant indirect of HGP on performance through self-efficacy efficacy (β = −0.05, SE = 0.04, *p* > 0.05). These results show that self-efficacy mediates the direct effect of PKMT on performance while that of HGP on performance is not mediated, beyond chances. This finding confirms, in part, the plausibility of Hypothesis three. Finally, the significant standardized residual estimate of 0.70 on the Exam variable in Figure 2 shows that 30% of the variability in students’ performance is explained by both the prior mathematics knowledge and self-efficacy. This variability is considered to be moderately high, and more discussion about this is presented in the next section.

## Discussion, Limitations, and Recommendations

### Discussion of Findings

Self-efficacy has been articulated theoretically to be an important construct in explaining variability in students’ performance. Several pieces of empirical evidence have demonstrated its relevance to students’ performance in psychology, sport, and clinical medicine (Bandura, 1997). Meanwhile, due to the task-specificity of self-efficacy, it could be erroneous to assume generalization of findings from other fields to the mathematics learning context. More so, there are limited studies with a focus on mathematics self-efficacy and its effects on students’ performance in university mathematics. As such, attempts are made in the present study to investigate the causal effects of mathematics self-efficacy on students’ performance through an innovative approach of instrumental variable modeling (Greenland, 2000). Prior mathematics knowledge (PKMT and HGP) and self-efficacy (CSEI) are conceptualized and operationalized based on previous studies and the self-efficacy theory. The measurement model of PKMT was evaluated, and it was found to provide reliable estimates of the construct it was hypothesized to expose. The findings of the present study also confirm reliable estimates of the measurement model of CSEI. These findings are consistent with the findings of previous studies on the two measures (Zakariya et al., 2019, 2020a). After establishing acceptable measurement models of the two measures, the hypothesized structural relationship between the research constructs was evaluated. The major findings are discussed in the forthcoming paragraphs.

The results of the present study confirm a direct effect of prior mathematics knowledge test on students’ calculus self-efficacy. This finding can be interpreted to mean that students with high scores on the prior mathematics knowledge test have a high sense of self-efficacy in solving first-year calculus tasks successfully. This finding is consistent with the postulated impact of personal previous task-based achievement on self-efficacy by the self-efficacy theory (Bandura, 2012). It was found that prior mathematics knowledge test alone accounts for 27% (i.e., the square of 0.52 times 100%) of the variability of the self-efficacy. However, this percentage of explained variance reduced to 24% when this direct effect of the test scores is combined with the direct effect of HGP on self-efficacy. The direct effect of prior knowledge of mathematics test on self-efficacy found in the present study is far higher than the effects of high school level, and the college credits (both operationalized to measure prior experience) on students’ self-efficacy in completing mathematics problem-solving tasks reported, elsewhere (Pajares and Miller, 1994; Pajares and Kranzler, 1995). Given that these studies are relatively old and the mathematics curriculum in higher education is changing to catch up with our 21st-century challenges, it is claimed that the present finding is novel and the captures current situation on the causal relation between prior mathematics knowledge and self-efficacy among university students.

Another major finding of the present study is the exposed direct effect of calculus self-efficacy on students’ performance in the course. A unique feature about the estimate of this direct effect lies in the ability of the instrumental variable approach to discern this effect from that of other disturbances which affect students’ performance but are not included in the model. This finding is interpreted to mean a high sense of self-efficacy is a potential cause of high scores of students, beyond chances, in the first-year introductory calculus course. By implication, this finding provides empirical support for designing interventions that foster self-efficacy as proxies to enhance students’ performance in the first-year introductory mathematics course. Such interventions may be in the inform of realistic modeling of the links between previous achievements and self-efficacy, social persuasion by older students who have passed the course, and other related activities that can be traced to the sources of self-efficacy. The magnitude of the estimated causal effect of self-efficacy on students’ performance in the present study is substantially higher than comparable direct effects reported in previous studies (Pajares and Kranzler, 1995; Roick and Ringeisen, 2018). As such, the author claims that the causal relationship exposed between self-efficacy and performance by the findings of the present study has a significant contribution to mathematics education literature.

Apart from the substantial contribution of the calculus self-efficacy to students’ performance exposed in the present study, a major finding is the detected mediating role of self-efficacy between prior knowledge mathematics test and students’ current performance in the course. It was found in the present study that about 46% (i.e., 0.17 out of 0.37) of the total effect of prior mathematics knowledge (PKMT and HGP) on students’ performance is mediated by self-efficacy. On the one hand, this finding may be interpreted to mean students with high scores on both the prior knowledge of mathematics test and the self-efficacy performed, beyond chances, better than the students who do not score high on the two measures. On the other hand, it confirms the mediating role of self-efficacy as postulated by the self-efficacy theory (Bandura, 1997). This finding also corroborates the mediating role of mathematics self-efficacy that is reported, elsewhere, using path analysis (Pajares and Miller, 1994). Despite the limited number of variables the author considered in the evaluated structural model of the relationship between the research constructs, the percentage of the explained variance (30%) in students’ performance is higher than the reported values in studies with several predictor variables (Pajares and Miller, 1994, 1995). It is conjectured that the task-specificity of the self-efficacy measure coupled with the innovative instrumental variable approach used in the present study contributes to the moderately high percentage of explained variance in the students’ performance. Potential variables that could increase the percentage of explained variance, if included in the model, are approaches to learning mathematics, academic motivation, mathematics anxiety, and attitudes toward mathematics learning. Future studies are recommended with this intention.

### Potential Limitations and Recommendations

A potential limitation of the present study is attributable to the restriction of sample to first-year engineering students enrolled on a course. Even though this restriction offers several advantages as previously highlighted in the “Materials and Methods” section, it might also hinder the generalization of the findings beyond a similar student population. Future replicated studies are recommended with a focus on students following a variety of courses at different levels of higher education. However, such studies should devise innovative ways or use robust statistical modeling such as multi-level SEM combined with the instrumental variable approach to account for task-specificity of the self-efficacy across diverse populations. Also, the relatively small sample size (189 students) could be a threat to the validity of the SEM results given that some researchers have recommended higher sample sizes (Marsh et al., 1998; Byrne, 2012). However, it has been theoretically argued and empirically shown that a “one size fits all” rule is not tenable for sample sizes of SEM studies (Wolf et al., 2013). As such, sample sizes close to 200 cases are recommended for conducting SEM studies that involve moderately complex models (Kline, 2016). Notwithstanding, future replication studies are recommended with a larger sample size to cross-validate the findings of the present study.

More so, the self-efficacy theory postulates a feedback causal relationship between self-efficacy and students’ performance in mathematics through reciprocal determinism model (Borgonovi and Pokropek, 2019). Nevertheless, the focus of the present study is only on one-directional causal effect from self-efficacy to students’ performance which could also constitute a limitation. The author argues that such a feedback causal relationship is better investigated using a longitudinal research design (e.g., Roick and Ringeisen, 2018) than the survey research design used in the present study. As such, future longitudinal studies are recommended with this intention. The author also acknowledges that a limited number of predictor variables in the evaluated structural model of the present study may constitute another limitation. Had been more relevant variables such as approaches to learning, motivation, and mental ability that have been linked with performance are included in the model (Pajares and Miller, 1994; Zakariya et al., 2020b), the percentage of explained variance in students’ performance would have improved. Future study may also be conducted with this intention.

## Conclusion

The present study is motivated by the lack of empirical evidence on the causal relationship between self-efficacy and students’ previous and current performance in university mathematics. Therein, attempts are made to fill this gap by investigating hypothesized causal claims between the research constructs using the instrumental variable approach to modeling. The major findings in the present study establish a causal relationship with reliable estimates between self-efficacy and students’ performance in an introductory calculus course at a university in Norway. The author conjectures that these findings are generalizable to similar student populations within and beyond Norwegian borders. This conjecture is based on both theoretical and innovative statistical perspectives adopted in the present study. As such, the author recommends replication of the present study to investigate this conjecture within the quantitative research paradigm. The author declares that an outright discovery of the causal relationship between self-efficacy and students’ performance in mathematics is not claimed in the present study. Instead, it is hoped that foundations are laid for future experimental, randomized-control trial studies with this intention.

## Data Availability Statement

The raw data supporting the conclusions of this article will be made available by the authors, without undue reservation.

## Ethics Statement

The studies involving human participants were reviewed and approved by Norwegian Centre for Research Data. The patients/participants provided their written informed consent to participate in this study.

## Author Contributions

YZ: conceptualization, methodology, formal analysis, software, data curation, investigation, visualization, and writing-original draft preparation.

## Conflict of Interest

The author declares that the research was conducted in the absence of any commercial or financial relationships that could be construed as a potential conflict of interest.

## Figures and Tables

**FIGURE A1:**
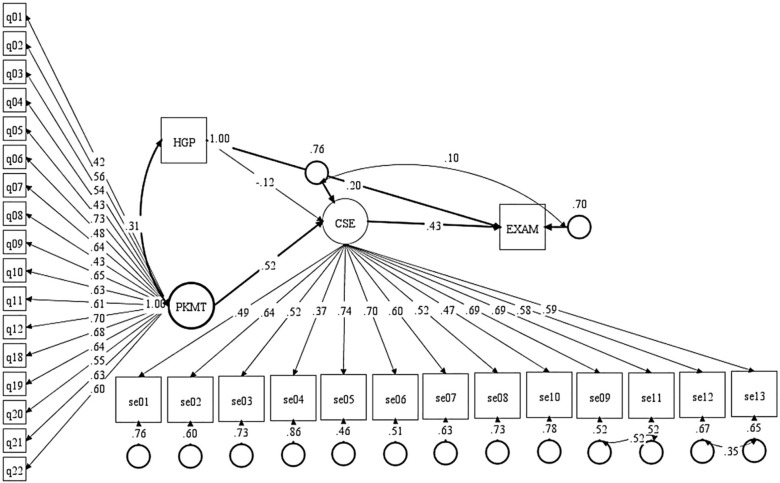
The full evaluated model of the relationship between prior mathematics knowledge, self-efficacy, and students’ performance in an introductory calculus course. The significant paths are in bold faces.
